# Multiple non-polypoid mucosal Schwann cell hamartomas presenting as edematous and submucosal tumor-like lesions: a case report

**DOI:** 10.1186/s12876-021-01607-w

**Published:** 2021-01-19

**Authors:** Takeshi Okamoto, Takaaki Yoshimoto, Katsuyuki Fukuda

**Affiliations:** Department of Gastroenterology, St. Luke’s International Hospital, 9-1 Akashicho, Chuo-ku, Tokyo, 104-8560 Japan

**Keywords:** Polyp, Neurogenic tumor, Harmatomatosis, Colonoscopy, Case report

## Abstract

**Background:**

Mucosal Schwann cell hamartomas are rare neurogenic tumors which present most commonly in the distal colon. They are usually discovered as small, single polyps in asymptomatic patients.

**Case presentation:**

An asymptomatic 64-year-old man was referred to us after a 12 mm subepithelial lesion was discovered incidentally on screening colonoscopy. Follow-up colonoscopy conducted 2 months later revealed that the tumor had disappeared, leaving multiple edematous, submucosal tumor-like elevations presenting as skip lesions throughout the sigmoid colon. Lesions had elongated pits on magnifying endoscopy and were limited to the first layer on endoscopic ultrasound. Biopsies revealed unclearly delineated foci of spindle-shaped cells limited to the lamina propria. On immunohistochemistry, all lesions were positive for S-100 protein and negative for CD34, CD56, and neurofilament protein. The patient was diagnosed with multiple mucosal Schwann cell hamartomas of the sigmoid colon. He remains asymptomatic after 18 months of follow-up, but the lesions have also remained unchanged.

**Conclusion:**

We report a case of multiple non-polypoid mucosal Schwann cell hamartomas. Endoscopic findings may assist in the differential diagnosis, particularly when presenting as non-polypoid, submucosal tumor-like lesions.

## Background

Mucosal Schwann cell hamartomas (MSCH) are rare neurogenic lesions found in the lamina propria of the gastrointestinal tract. They arise most commonly in the distal colon and are generally asymptomatic small, single sessile polyps which may be discovered incidentally on screening colonoscopy [1]. MSCH has also been reported in the gastroesophageal junction [2], gastric antrum [3], and gallbladder [4].

The differential diagnosis of neurogenic tumors in the colon includes mucosal neuroma which is associated with multiple endocrine neoplasia type 2B (MEN 2B), neurofibroma which is associated with neurofibromatosis type 1 (NF1), schwannoma, ganglioneuroma, intramucosal perineuroma, and mucosal benign epithelioid nerve sheath tumor [1, 2]. MSCH can be distinguished histologically, by proliferation of uniform spindle cells with unclear cell borders and dense eosinophilic cytoplasm and elongated nuclei in the lamina propria which entrap colonic crypts, and on immunohistochemistry, with Schwann cells positive for S-100 protein, generally negative for neurofilament protein (NFP), and negative for other stains including CD34, smooth muscle actin, and epithelial membrane antigen [1, 2, 5, 6].

While there are currently over 80 reports of colorectal MSCH in the English literature, reports of non-polypoid MSCH and of multiple MSCH are scarce [2, 7]. Furthermore, endoscopic findings in MSCH have not been reported in detail, as most authors have focused on its pathological aspects. Herein, we report a case of multiple non-polypoid, edematous and submucosal tumor-like MSCH spanning most of the sigmoid colon discovered incidentally on screening colonoscopy. We also consider the narrow-band imaging (NBI) and endoscopic ultrasound findings of MSCH.

## Case presentation

**Fig. 1:**
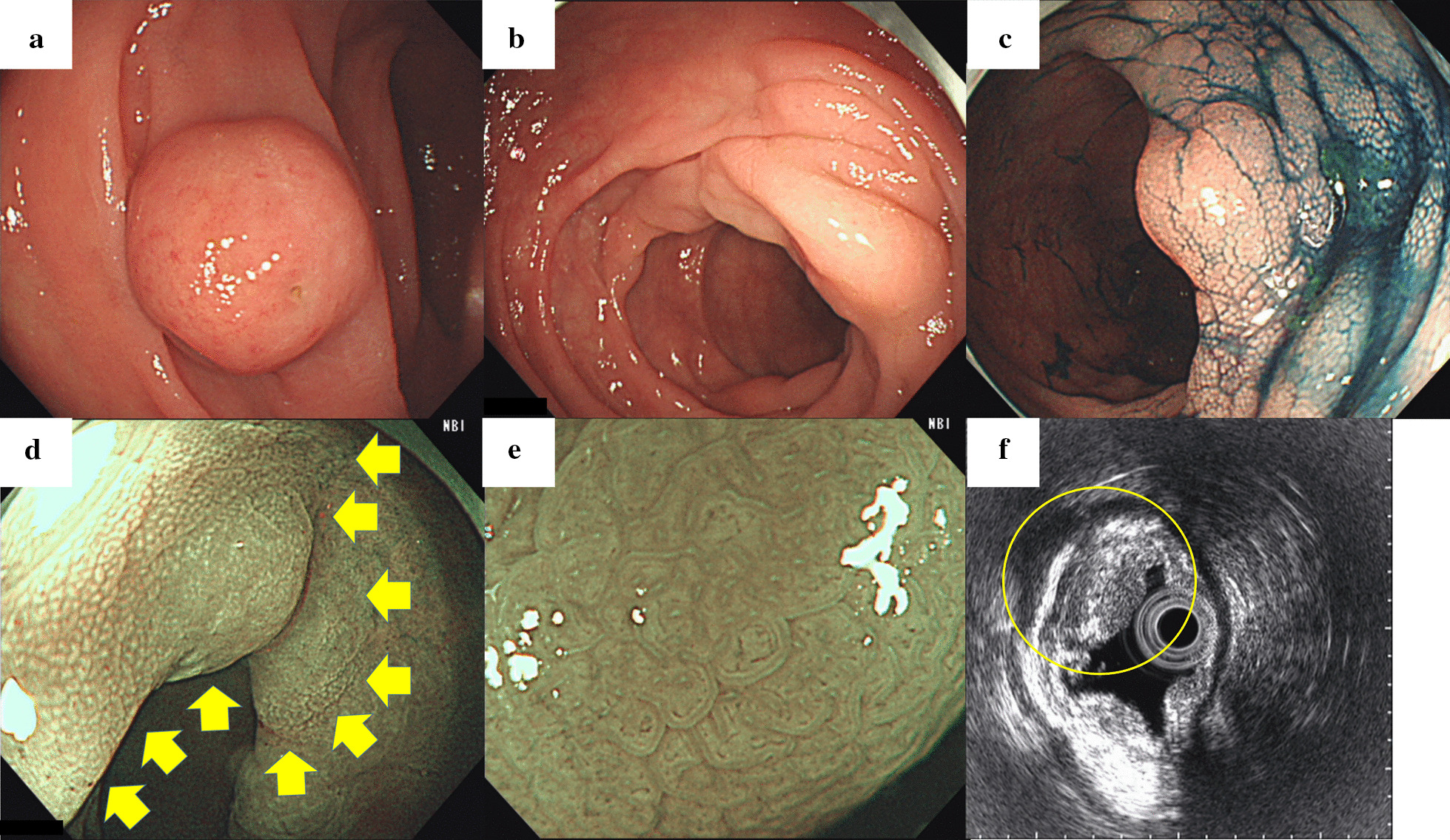
**a** A subepithelial lesion was observed on initial colonoscopy but subsequently disappeared. **b** Mucosal Schwann cell hamartomas presenting as numerous edematous elevations and submucosal tumor-like projections were observed throughout the sigmoid colon. **c** Indigo carmine clearly delineates a submucosal tumor-like projection. **d** Narrow-band imaging (NBI) of a Schwann cell hamartoma with fine white granular opacities on its surface (yellow arrows). **e** Magnifying narrow-band imaging revealed elongated crypt openings, increased width of intervening parts, and no visible microvessels. **f** Endoscopic ultrasound revealed mild, uniformly hypoechoic thickening limited to the first layer (yellow circle)

A 64-year-old man was referred to our hospital after a 12 mm subepithelial lesion in the sigmoid colon was discovered incidentally on screening colonoscopy (Fig. 1a). The referring physician’s biopsy report stated that only non-specific mucosal changes were observed. The patient had a history of hypertension treated with calcium channel blockers and angiotensin II receptor blockers, type 2 diabetes mellitus treated with a selective dipeptidyl peptidase-4 inhibitor, and atrial fibrillation treated with a direct oral anticoagulant. He was in his ordinary state of health, had regular bowel movements, and denied any abdominal symptoms.

**Fig. 2:**
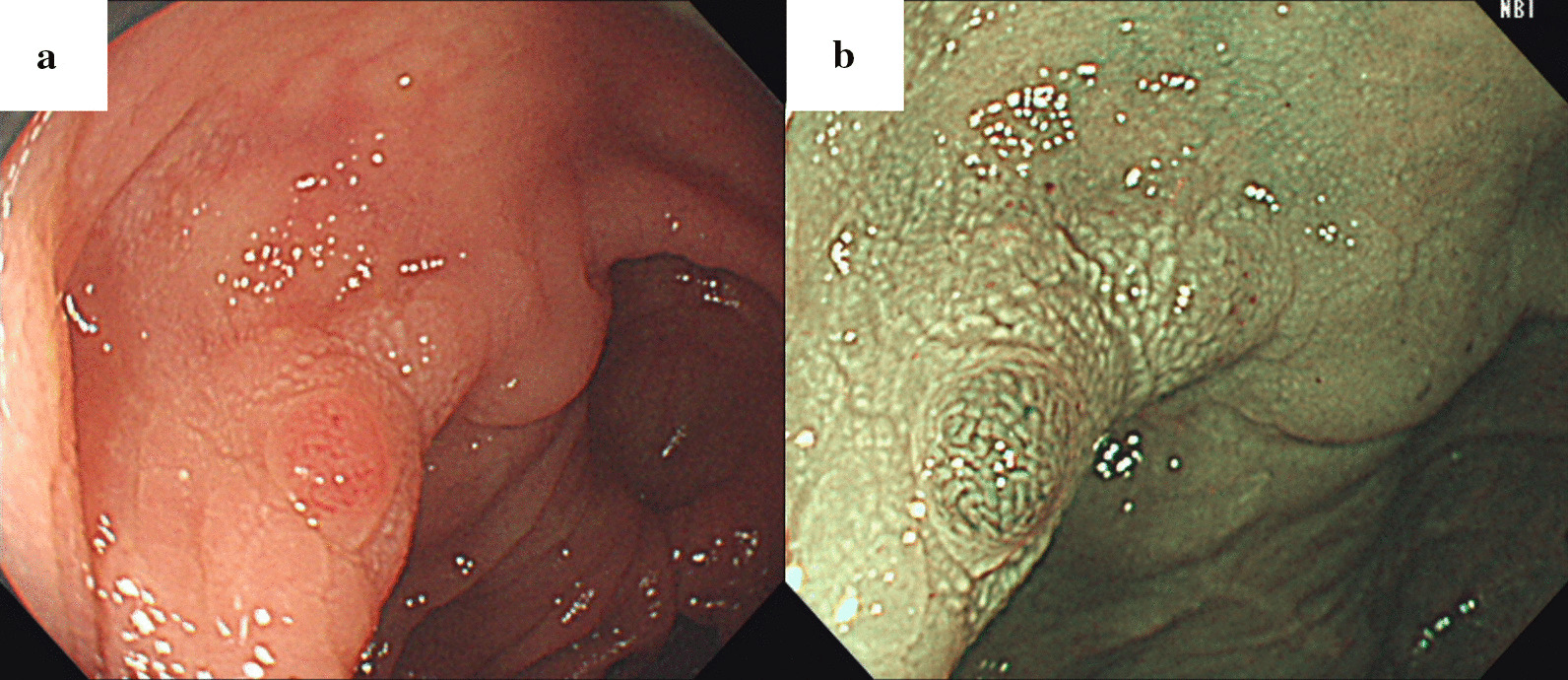
**a** A hyperplastic polyp was located on top of one of the mucosal Schwann cell hamartomas elevated lesions with white granular opacities. **b** Narrow-band imaging of the hyperplastic polyp and mucosal Schwann cell hamartoma

Follow-up colonoscopy conducted 2 months later revealed that the tumor had disappeared completely, leaving numerous edematous, submucosal tumor-like protrusions presenting as skip lesions throughout the sigmoid colon, over a length of 15 cm (Fig. 1b, c). Some lesions had fine white granular opacities on their surface on NBI, while others did not (Fig. 1d). All lesions displayed elongated crypt openings, increased width of intervening parts, and no visible microvessels on magnifying NBI (Fig. 1e). Endoscopic ultrasound revealed mild, homogeneously hypoechoic thickening of the first layer (superficial mucosa) with no apparent second layer (deep mucosa) involvement (Fig. 1f). Six tubular adenomas (4 in the transverse colon, 2 in the sigmoid colon), one serrated adenoma in the sigmoid colon, and four hyperplastic polyps in the sigmoid colon were resected during the same session. One of the resected hyperplastic polyps in the sigmoid colon was located on top of one of the elevated lesions with white granular opacities (Fig. 2a, b). Computed tomography with contrast was unremarkable, confirming no clear tumor or thickening of the colonic wall.

**Fig. 3:**
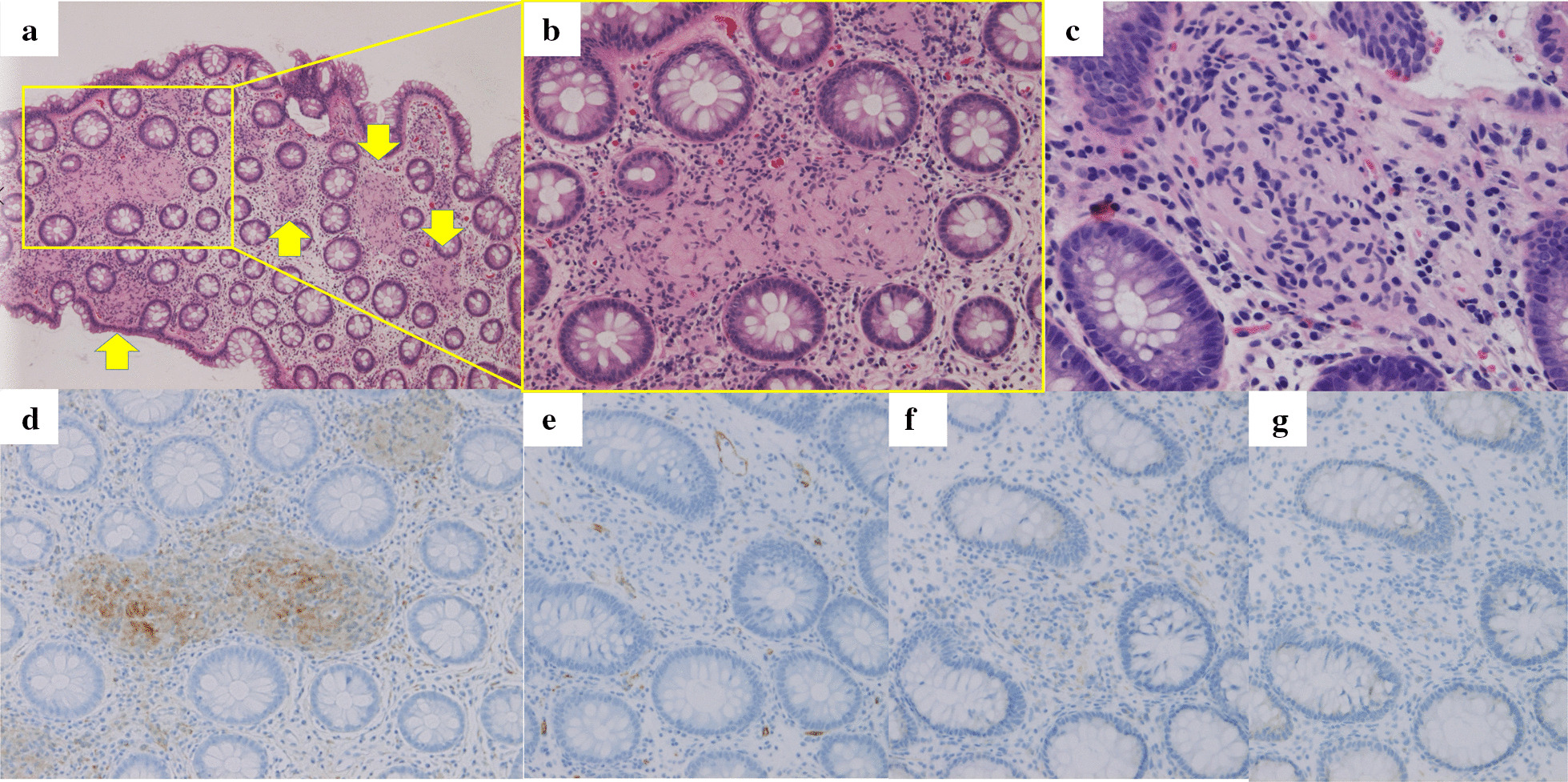
**a** Low-power and **b**, **c** high-power magnification of mucosal Schwann cell hamartomas (yellow arrows). Immunohistochemistry: **d** S-100 protein, **e** CD34, **f** CD56, and **g** neurofilament protein stains

Multiple biopsies of the thickened mucosa all revealed proliferation of uniform spindle-shaped cells in the lamina propria (Fig. 3a–c). Eosinophilic cytoplasm with unclear cell borders and tapered or elongated nuclei were observed. Crypts and goblet cells were preserved. Edema and mild lymphocytic and plasma cell infiltration were observed in the interstitium. Schwann cells positive for S-100 protein and negative for CD34, CD56, and NFP were identified on immunohistochemistry (Fig. 3d–g). Stains for pan-cytokeratin (AE/AE3) and desmin were also negative, as were chromogranin A and synaptophysin stains conducted on biopsies which included submucosal tissue.

Upon detailed questioning, the patient denied any personal or family history of café au lait spots, neurofibromatosis, or thyroid or adrenal disease which could suggest MEN 2B or NF1. No relevant findings were found in a dermatological examination, laboratory tests, or in imaging studies. Based on the above, the patient was diagnosed with multiple MSCH of the sigmoid colon.

The patient remains asymptomatic without treatment during 18 months of follow-up. However, the multiple MSCH lesions in the sigmoid colon remain unchanged despite frequent colonoscopies. Pathological findings consistent with MSCH are also observed on follow-up biopsies taken at every colonoscopy.

## Discussion and conclusion

Gibson et al. [1] first reported 26 cases of MSCH presenting as sessile polyps 1–6 mm in diameter, most of which were asymptomatic. Subsequent reports were limited to single cases until Li et al. [2] reported 48 MSCH cases of the colon as well as 6 MSCH cases of the gastroesophageal junction. The authors also summarized the 85 reported cases of MSCH in the English literature. In the 86 cases including ours, the average age was 60.2 years and 47.7% were male. Most were found distal to the splenic flexure (77.9%), particularly the sigmoid colon (47.7%). While 81.0% were undergoing screening colonoscopy, other indications for colonoscopy included bleeding, diarrhea or loose stools, abdominal pain, and constipation (Table 1).

**Table 1:** Summary of reported colorectal cases of mucosal Schwann cell hamartoma (n = 86)

	n	%
*Location*		
Appendiceal orifice	3	3.5
Cecum	2	2.3
Ascending colon	7	8.1
Transverse colon	7	8.1
Descending colon	16	18.6
Sigmoid colon	41	47.7
Rectosigmoid colon	2	2.3
Rectum	8	9.3
*Indication/symptoms*		
Screening	67	77.9
Bleeding	8	9.3
Diarrhea/loose stools	4	4.7
Pain	3	3.5
Constipation	1	1.2
Ulcerative colitis	1	1.2
Unknown	2	2.3

Bae et al. [7] reported the only case of multiple MSCH, referred to as mucosal Schwann cell hamartomatosis. In that case, colonoscopy conducted on a young man with abdominal discomfort and loose stools revealed a polyp in the mid-rectum and polyposis-like mucosal changes in the distal rectum. The mid-rectum polyp and one of 3 biopsies taken from polyposis-like mucosa revealed histological findings consistent with MSCH. On the other hand, biopsies were taken from 5 distinct mucosal lesions in our case, all of which were consistent with MSCH. The fact that our case was asymptomatic despite the large area of the colon occupied by MSCH may be interpreted as further indirect evidence that MSCH is harmless and does not cause symptoms. However, follow-up is desirable as long-term prognosis has not been documented.

There are only 3 other reports of non-polypoid MSCH. The first presented as many small whitish nodules somewhat similar to lesions found in our case, the second discovered incidentally on random biopsy conducted for evaluation of chronic diarrhea, and the third was located at the mass base following endoscopic mucosal resection [2, 8]. It is also interesting to note that a hyperplastic polyp was observed on top of a MSCH in our case, which has never been reported.

Details on endoscopic MSCH findings in the literature are scarce [8]. Elongated crypt openings, increased width of intervening parts, and no visible microvessels on magnifying NBI with or without white granular opacities were consistently observed in the multiple lesions of our case and may assist in the differential diagnosis during colonoscopy. Lesions were limited to the first layer on endoscopic ultrasound, which can clearly differentiate MSCH from submucosal tumors. This is also consistent with MSCH being localized in the lamina propria on histology. In cases where MSCH mimics submucosal tumors, this finding may help prevent unnecessary endoscopic or surgical resection.

The cause of the vanishing tumor identified by the referring physician remains a mystery. Reports of vanishing colonic tumors include cytomegalovirus infection, angioedema resulting from acquired type II C1-inhibitor deficiency, and colorectal cancer [9–11]. Vanishing tumor due to anisakiasis has been reported in the stomach and can most likely also occur in the colon [12]. Spontaneous regression of various tumors such as neuroendocrine tumors has also been reported, particularly after biopsy [13]. However, our patient was asymptomatic, was believed to be immunocompetent in spite of his well-controlled diabetes mellitus, and had no history of raw fish ingestion. The biopsy taken from the surface of the sigmoid tumor only revealed mucosal tissue, making it unlikely that a submucosal tumor existed and regressed spontaneously due to insult from the biopsy. While we can provide no evidence, the proximity of the vanished tumor to the MSCH lesions led us to suspect an association. More research is required to determine whether or not MSCH can present as a vanishing tumor.

In conclusion, we report a case of multiple MSCH presenting as edematous, submucosal tumor-like lesions in the sigmoid colon. NBI and endoscopic ultrasound findings may help the endoscopist to suspect atypical MSCH which does not take the form of a small sessile polyp. Discovery of similar cases may shed light on the pathogenesis of this seemingly harmless entity in the future.

## Data Availability

Not applicable.

## Abbreviations

MSCH: Mucosal Schwann cell hamartoma
MEN 2B: Multiple endocrine neoplasia type 2B
NF1: Neurofibromatosis type 1
NFP: Neurofilament protein
NBI: Narrow-band imaging
